# Prediction of Lymphovascular Invasion in Early–Stage Lung Adenocarcinoma Using Artificial Intelligence–Based Radiomics

**DOI:** 10.3390/cancers17243998

**Published:** 2025-12-15

**Authors:** Yoshihisa Shimada, Kazuharu Harada, Yujin Kudo, Jinho Park, Jun Matsubayashi, Masataka Taguri, Norihiko Ikeda

**Affiliations:** 1 Department of Thoracic Surgery, Tokyo Medical University, Tokyo 160–8402, Japan; 2 General Surgery Department, Sunrise Japan Hospital Phnom Penh, Phnom Penh 12301, Cambodia; 3 Department of Health Data Science, Tokyo Medical University, Tokyo 160–8402, Japan; haradak@tokyo-med.ac.jp (K.H.);; 4 Department of Radiology, Tokyo Medical University, Tokyo 160–8402, Japan; 5 Department of Anatomic Pathology, Tokyo Medical University, Tokyo 160–8402, Japan; jun-ma@tokyo-med.ac.jp

**Keywords:** lymphovascular invasion, AI, radiomics, extracellular vesicles, lung adenocarcinoma

## Abstract

**Objectives:** This study utilized artificial intelligence (AI)–based radiomics analysis of computed tomography (CT) images using a modified U–Net for lung nodule segmentation and convolutional neural network based on VGG–16 to predict lymphovascular invasion (LVI) in stage 0–I lung adenocarcinoma. Additionally, the study investigated whether combining radiomics data with serum microRNA (miR)–30d level as a potential biomarker could enhance predictive performance. **Methods:** A total of 1265 patients who underwent complete resection between 2008 and 2018 were included. AI–based CT analysis was performed, and logistic regression was applied to predict LVI using 35 imaging features. A risk score (RS) generated from 840 patients in the derivation cohort was used to identify a high–risk group, with validation performed using 425 patients. Additionally, 47 cases with extracellular vesicle (EV)–derived miR–30d level data were analyzed to evaluate the value of the integrated approach. **Results:** Among all the patients, 467 patients (36.9%) were LVI–positive, and LVI was independently associated with poorer overall survival. The receiver operating characteristic curve for LVI based on the RS yielded an area under the curve of 0.899. For LVI prediction, the sensitivity, specificity, and accuracy were 84.8%, 83.7%, and 83.9%, respectively, in the derivation group, and 82.3%, 79.4%, and 80.5%, respectively, in the validation group. The integrated approach with miR–30d enhanced the predictability of LVI, achieving a sensitivity of 93.3%, specificity of 70.5%, and accuracy of 85.1%. **Conclusions:** AI–based radiomics demonstrated high effectiveness for predicting LVI, with RSs showing broad clinical applications. The addition of EV–derived miR–30d modestly improved predictability.

## Simple Summary

The study developed an artificial intelligence (AI)–based radiomics model using a modified U–Net for lung nodule segmentation and a VGG–16–based convolutional network to predict lymphovascular invasion (LVI) in stage 0–I lung adenocarcinoma. Computed tomography (CT) data from 1265 patients were analyzed, and a risk score derived from 35 imaging features was validated in an independent cohort. Logistic regression identified LVI as an independent factor associated with poor prognosis. The receiver operating characteristic curve for LVI prediction based on this risk score yielded an area under the curve of 0.899. The sensitivity, specificity, and accuracy were 84.8%, 83.7%, and 83.9%, respectively. AI–based radiomics demonstrated high effectiveness for predicting LVI, and the risk score may have broad clinical applications.

## 1. Introduction

Pathological lymphovascular invasion (LVI), defined as the presence of blood vessel invasion (BVI) and/or lymphatic permeation (Ly), is recognized as a potent and independent prognostic indicator across various malignancies, including gastric, urothelial, colorectal, esophageal, cervical, breast, and early–stage non–small cell lung cancers (NSCLCs) [1,2,3,4,5,6,7,8,9]. LVI represents tumor aggressiveness and constitutes a pivotal event in the cascade of invasion and metastasis [1,10,11,12]. Ma et al. demonstrated that most lung adenocarcinomas ≤ 2 cm can be effectively treated with wedge resection, while in the case of lung adenocarcinomas with BVI undergoing wedge resection, the clinical outcomes were poor, with recurrence rates of 40% to 45% within 5 years and overall mortality reaching 60% to 65% at 7 years [13]. Thus, the presence of LVI may critically inform surgical decision–making in early–stage lung cancer. The negative impact of LVI on recurrence and prognosis has been well demonstrated, and several preoperative factors—such as sex, smoking history, tumor size, tumor markers, and histological subtype—have been proposed as potential predictors [1,2,14,15]. Nevertheless, reliable non–invasive methods for the preoperative diagnosis of LVI remain unavailable.

A growing body of research has explored radiomics approaches based on various imaging modalities, such as computed tomography (CT) and positron emission tomography (PET), often integrated with artificial intelligence (AI) techniques, across multiple cancer types. In early–stage NSCLC, this strategy has facilitated automated three–dimensional (3D) assessments of lung nodules, offering insights into prognosis, recurrence, and unfavorable biological behavior [16,17,18,19]. Radiomics, which transforms medical images into large–scale, predefined quantitative datasets, holds great promise. In particular, CT–based radiomics could serve as a useful tool for predicting early postoperative recurrence and lymph node metastasis, though its clinical utility remains to be fully established [16,18,19,20].

Moreover, a novel and specific microRNA (miRNA) marker, miR–30d, was identified as being associated with LVI through small RNA sequencing of serum–derived extracellular vesicles (EVs) in patients with early–stage lung adenocarcinoma [21]. EVs are bioactive vesicles that facilitate cell–to–cell communication by shuttling their cargos, including miRNAs, mRNAs, proteins, and lipids [22,23,24,25,26].

To date, no studies have investigated the prediction of LVI in lung cancer using radiomics analysis based on large–scale imaging datasets. Moreover, no previous studies have combined two noninvasive approaches—for example, imaging–based radiomics and liquid biopsy—for predictive analysis. The development of reliable noninvasive methods for predicting LVI remains an important unmet clinical need. The primary objective of this study is to evaluate the ability of AI–based radiomics analysis of preoperative CT scans to predict LVI in patients with early–stage lung adenocarcinoma. Additionally, the predictive performance of a combined model integrating AI–based radiomic features and serum EV–derived miR–30d expression was assessed in a subset of these patients.

## 2. Materials and Methods

### 2.1. Ethical Statement

This study was approved by the Institutional Review Board of Tokyo Medical University (IRB No. SH3951, 26 December 2017). Written informed consent for the use and analysis of clinical data was obtained from each patient prior to the surgery.

### 2.2. Patients

**Figure 1:**
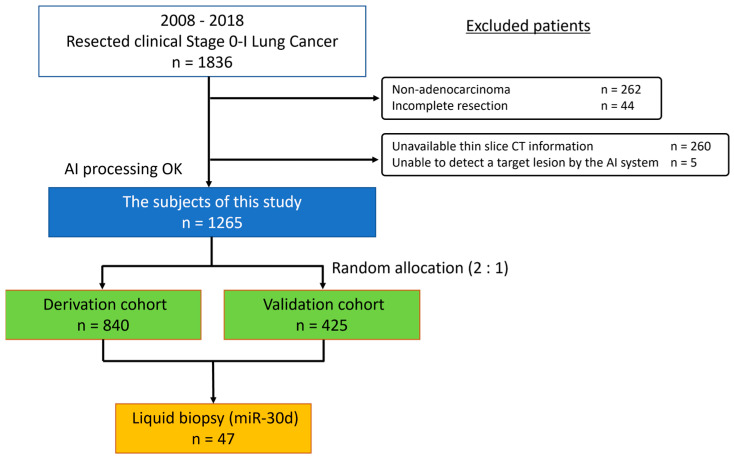
Consort diagram of patients included in the study. CT, computed tomography; AI, artificial intelligence.

A total of 1836 patients diagnosed with clinical stage 0–I lung cancer who underwent pulmonary resection at Tokyo Medical University Hospital between January 2008 and December 2018 were initially reviewed. Patients were excluded if they had non–adenocarcinoma histology, underwent incomplete surgical resection, lacked thin–slice CT data, or if the AI algorithm failed to identify the target lesion. After applying these criteria, 1265 patients with lung adenocarcinoma were selected for the study. To minimize overfitting, the dataset was randomly divided into independent derivation (n = 840) and validation (n = 425) cohorts using computer–generated randomization without considering clinical variables to avoid selection bias. Model performance in the validation cohort was monitored throughout model development to evaluate generalizability. A consort diagram of the patients included in this study is shown in Figure 1. The medical records of all patients were reviewed, including the TNM stage, which was determined according to the 8th edition of the TNM classification of malignant tumors.

### 2.3. Radiological Evaluation of Primary Tumor

All study participants underwent high–resolution CT before surgery. Helical CT images (1.25 mm thick) were obtained from the whole lung. Chest CT performed within one month prior to surgery was routinely used for preoperative assessment. If the interval between the latest CT and surgery exceeded two months, a repeat CT scan was conducted on the day before the operation. The total tumor and solid part sizes were preoperatively measured by an experienced thoracic radiologist (J.P) and a thoracic surgeon (Y.S). The size of the solid part was defined as the maximum dimension of the solid component of the lung window, excluding ground–glass nodules. PET/CT was routinely introduced for preoperative staging after 2011; however, it was generally not performed in patients with severe diabetes mellitus, a history of contrast agent allergy, or those who declined the examination. During the study period, the institution where the PET examinations were conducted was changed, and scans were performed at three different facilities. Therefore, standardization of the quantitative metrics across these institutions was considered difficult.

### 2.4. Radiomics and AI Imaging Analysis

After Digital Imaging and Communications in Medicine data were imported into the Synapse Vincent platform (Fujifilm Corporation, Tokyo, Japan), the integrated AI software (Beta Version; Fujifilm Corporation) automatically identified and segmented pulmonary nodules, generating 3D reconstruction of the lungs and nodules. The segmentation process was implemented using a 3D convolutional neural network based on a modified U–Net architecture comprising 17 convolutional layers. The algorithm distinguishes the solid component of each nodule from the nonsolid part, and calculates parameters such as size, volume, solid–to–nonsolid ratio, total lesion volume, and CT histogram features. Among the 39 imaging features, four were initially excluded from the analysis because they exhibited high similarity in image characteristics, potentially leading to statistical interference during the scoring process. Consequently, a total of 35 AI–derived imaging features were evaluated, comprising 15 features from lung nodule analysis (including nodular area, volume, length, and CT histogram parameters) and 20 radiomics features. The radiomics features were automatically extracted and displayed as a score from 0 to 1 using the feature analysis function.

### 2.5. AI Architecture for Nodule Segmentation and Feature Extraction

For automated lung nodule segmentation, the AI system employed a modified 3D U–Net architecture. U–Net is an encoder–decoder convolutional neural network (CNN) designed for medical image segmentation, which consists of a contracting path that captures contextual information and an expanding path that restore special resolution. The AI–based lung nodule analysis model was built using a CNN based on the VGG–16 architecture. It consists of 12 convolutional layers, with four layers removed from the output side of the original VGG–16. Model parameters were optimized according to standard training practices and previously established settings to ensure stable convergence and reproducibility. To extract 3D radiomics features, 3D convolution was applied to all convolution layers. The VGG–16 framework was selected for its proven performance and interpretability in medical image analysis tasks. Because the AI system used in this study was developed as an integrated, proprietary software module by the manufacturer, detailed training parameters were not accessible to the authors. The segmentation and feature–extraction models operated under predefined and validated internal parameters established by the developer to ensure consistent and reproducible performance across all cases.

### 2.6. The Risk Score for LVI

A logistic regression model was established for the risk score for LVI based on the 35 AI imaging features. Before performing logistic regression, 20 radiomics features were pre–processed (Table S1). Some features exhibited a skewed distribution concentrated near 0 or 1. When following such distributions, there are issues known as ceiling and floor effects, in which differences near 0 or 1 are not properly evaluated. Therefore, an empirical logit transformation was performed to understand the radiomics features (Figure S1) [27]. The empirical logit transformation is a modified version of the logit transformation, represented by the following equation that includes adjustments for cases where the variable x takes exact values of 0 or 1.

logit (*x*) = log (*x*/1 − *x*), *x*: one of the radiomic features

The next step was to construct a risk score for LVI using all 35 AI imaging features including 20 transformed radiomics features. We calculated the prediction probabilities with respect to LVI.

### 2.7. Histopathology

All the surgical specimens were fixed in 10% formalin and embedded in paraffin. LVI was defined as the presence of either Ly or BVI. The presence of BVI was determined by identifying conspicuous clusters of intravascular cancer surrounded by an elastic layer. Ly was determined to be present when tumor cells floating in lymphatic vessels with no supporting smooth muscles or elastic fibers were identified. The pathological evaluation was reviewed by a single pathologist and one or more pathologists confirmed the diagnosis.

### 2.8. Isolation of EVs and Measurement of the miR–30d Level

Blood samples were collected from 47 patients before surgery. A sequential centrifugation procedure was performed to recover the EVs. The venous blood from each patient was separated into serum and cellular fractions. Cells were pelleted by centrifugation at 300× *g* for 5 min, followed by centrifugation at 1200× *g* for 20 min. To eliminate other cellular debris, the supernatant was centrifuged at 10,000× *g* for 30 min. For EV preparation, the samples were ultracentrifuged at 100,000× *g* for 35 min at 4 °C. The pellets thus obtained were then washed with phosphate–buffered saline.

RT–qPCR analysis was performed to quantify EV–derived miR–30d. The miRNAs were isolated using the miRNeasy Mini Kit and cDNAs was generated using the TaqMan MicroRNA Reverse Transcription Kit (Thermo Fisher Scientific, Waltham, MA, USA). Gene–specific TaqMan MicroRNA Probes (Thermo Fisher Scientific) were used for quantitative analyses of the miRNA transcript levels of miR–30d.

### 2.9. Statistics

Overall survival (OS) was measured from the date of surgery to the date of death from any cause or the date on which the patient was last known to be alive. Recurrence–free survival (RFS) was measured as the interval between the date of surgery and the date of recurrence, date of death from any cause, or date on which the patient was last known to be alive. Survival curves were plotted using the Kaplan–Meier method, and differences were tested using the log–rank test. Univariate and multivariate analyses were performed to identify factors associated with poor survival and LVI. A multivariate logistic regression analysis was performed to build a risk score. Pearson’s chi–square test and Student’s *t*–test were used to compare the two groups of data. Receiver operating characteristic (ROC) curves for LVI were constructed, and the optimal cutoff values were determined using the area under the curve (AUC). To evaluate the performance of the parameters in predicting LVI, we calculated the sensitivity, specificity, accuracy, positive predictive value (PPV), and negative predictive value (NPV). The AUC, sensitivity, specificity, accuracy, PPV, and NPV showed that the model provided high discriminative ability and reliable performance in predicting LVI. Patients in the derivation cohort were dichotomized at a risk score of 0.397, and exhibited a sensitivity of 84.8% and 82.3%, specificity of 83.7% and 79.4%, accuracy of 83.9% and 80.5%, positive predictive value (PPV) of 74.9% and 70.3%, and negative predictive value (NPV) of 90.4% and 88.3%. For the EV–miR–30d subgroup, 95% confidence intervals (CIs) for sensitivity, specificity, and accuracy were calculated as binomial proportions using the Clopper–Pearson exact method. All tests were two–sided, and statistical significance was set at *p* < 0.05. The Statistical Package for the Social Sciences (SPSS) software package (version 28.0, DDR3 RDIMM; SPSS Inc., Chicago, IL, USA) was used for statistical analysis. Kaplan–Meier curves were constructed using the R package (version 4.0.5).

## 3. Results

Patient characteristics are presented in Table 1. The median follow–up times for survivors in the derivation and validation cohorts were 1789 and 1798 days, respectively, with no significant differences between the two cohorts. Among all the patients, 467 patients (36.9%) were LVI–positive and 873 patients (69.0%) were preoperatively and pathologically diagnosed with lung cancer. The primary tumor was located in the right upper lobe in 439 patients (34.7%), right middle lobe in 83 (6.6%), right lower lobe in 244 (19.3%), left upper lobe in 312 (24.7%), and left lower lobe in 187 patients (14.8%). A total of 1250 (98.8%) nodules were peripherally located. Serum liquid biopsy was performed in 47 patients (3.7%).

**Figure 2:**
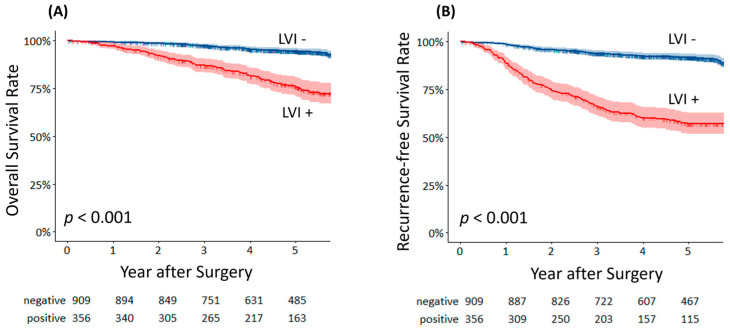
(**A**) Overall survival curves of lymphovascular invasion–positive and lymphovascular invasion–negative patients. (**B**) Recurrence–free survival curves of lymphovascular invasion–positive and lymphovascular invasion–negative patients.

Univariate and multivariate analyses for OS and RFS were conducted. Age (odds ratio (OR): 1.031; 95% confidence interval (CI): 1.011–1.051; *p* = 0.002), smoking status (OR 1.774; 95%CI 1.255–2.507; *p* = 0.001), comorbidities (OR: 1.515; 95%CI: 1.061–2.164; *p* = 0.022), surgical procedure (OR: 1.880; 95%CI: 1.151–0.067; *p* = 0.012), lymph node metastasis (OR: 2.943; 95%CI: 2.034–4.258; *p* < 0.001), and LVI (OR: 3.850; 95%CI: 2.564–5.782; *p* < 0.001) were independently associated with poorer OS (Table S2). Male sex (OR: 1.580; 95%CI: 1.221–2.045; *p* < 0.001), solid–part size (OR: 1.501; 95%CI: 1.215–1.853; *p* < 0.001), surgical procedure (OR: 1.770; 95%CI: 1.105–2.833; *p* = 0.018), lymph node metastasis (OR: 3.579; 95%CI: 2.674–4.789; *p* < 0.001), and LVI (OR: 3.382; 95%CI: 2.369–4.827; *p* < 0.001) were independently associated with poorer RFS (Table S3). Kaplan–Meier curves demonstrated that OS and RFS were significantly worse in patients with LVI than in those without LVI (Figure 2A, *p* < 0.001 and Figure 2B, *p* < 0.001). Ly was also a significant prognostic factor for OS (Figure S2A, *p* < 0.001) and RFS (Figure S2B, *p* < 0.001), and BVI had a significant impact on both OS (Figure S2C, *p* < 0.001) and RFS (Figure S2D, *p* < 0.001).

**Figure 3:**
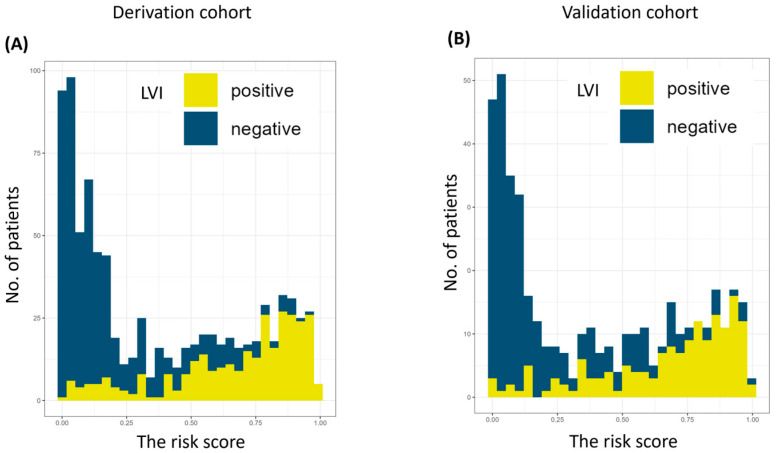
The number of patients according to risk score in the derivation cohort (**A**) and the validation cohort (**B**). AI, artificial intelligence. LVI, lymphovascular invasion.

Univariate and multivariate logistic regression analyses were conducted using all 35 AI–derived imaging features to build a risk score for predicting LVI. This analysis provided the odds ratios and *p* values for each parameter (Table S4). In the univariate analysis, all variables showed significant associations with LVI. The distributions of the calculated risk scores in the derivation and validation cohorts are shown in Figure 3, demonstrating a clear increase in the proportion of LVI–positive patients with higher risk scores. Furthermore, a heatmap (Figure S3) was used to visualize the relationships between the 35 AI imaging features and the presence of LVI, highlighting the significant features that contributed most to the prediction model.

To compare the risk score with other clinical factors associated with LVI, univariate and multivariate analyses were performed to identify factors associated with the presence of LVI among clinical variables, excluding the risk score, in patients assigned to the derivation cohort (Table S5). Multivariate analysis revealed that age (OR: 0.968; 95% C:I 0.951–0.986; *p* < 0.001), male sex (OR: 1.479; 95% CI: 1.048–2.088; *p* = 0.026), forced expiratory volume in 1 s as a percentage of the forced vital capacity (FEV1.0%) (OR: 1.479; 95%: CI 1.048–2.088; *p* = 0.026), and solid–part size (OR: 3.997; 95% CI: 3.159–5.058; *p* < 0.001), were independent significant factors for LVI.

Solid–part size was identified as the strongest factor associated with LVI among the clinical variables, excluding the risk score. Therefore, we compared the predictive performance of the risk score with that of solid–part size in identifying LVI. ROC curves were generated to evaluate the AUC and determine optimal cut–off values relevant to LVI. The risk score demonstrated an AUC of 0.899 with a cut–off score of 0.397 in the derivation cohort and an AUC of 0.882 in the validation cohort (Figure S4). In contrast, solid–part size showed an AUC of 0.803 with a cut–off size of 1.45 cm (Figure S5). These results suggest that the risk score is a highly accurate predictor of LVI, with superior performance metrics compared to solid–part size.

Detailed performance measures for the risk score in predicting LVI are shown in Table 2. Patients in the derivation cohort were dichotomized at a risk score of 0.397, and exhibited a sensitivity of 84.8% and 82.3%, specificity of 83.7% and 79.4%, accuracy of 83.9% and 80.5%, PPV of 74.9% and 70.3%, and NPV of 90.4% and 88.3% in the derivation and validation cohorts, respectively. With a solid–part size of 1.45 cm as the cut–off value, the analysis exhibited an AUC of 0.803 and 0.797, sensitivity of 83.1% and 81.6%, specificity of 65.9% and 66.7%, accuracy of 72.2% and 69.8%, PPV of 58.7% and 59.1%, and NPV of 87.1% and 86.0% in the derivation and validation cohorts, respectively (Table S6).

Our previous study demonstrated that serum miR–30d levels in EVs may serve as a predictive biomarker for detecting LVI in patients with early–stage lung adenocarcinoma. Therefore, we assessed the predictive performance of miR–30d for LVI in combination with the risk score to determine whether the prediction rate improved. This analysis used samples from 47 patients whose serum was collected preoperatively among the 1265 patients.

The cut–off level of 1.8 was determined as the median value for miR–30d levels. The results showed sensitivity, specificity, accuracy, PPV, and NPV values of 70.0% (95% CI: 45.7–88.1%), 82.4% (95% CI: 64.2–94.2%), 74.5% (95% CI: 59.7–86.1%), 87.5%, and 60.9%, respectively (Table S7). The risk score combined with a supplemental miR–30d level (miR–30d < 1.8 or risk score > 0.397) demonstrated a sensitivity of 93.3%, specificity of 70.5%, accuracy of 85.1%, PPV of 84.8%, and NPV of 85.7% (Table S8). The inclusion of miR–30d levels allowed for the detection of LVI in a few additional patients (Figure S6).

## 4. Discussion

LVI has significant prognostic implications in patients with early–stage lung cancer [1,2,3]. Local tumor invasion and the intravasation of tumor cells occur early in the metastatic cascade, which may explain why LVI is often observed in patients without lymph node metastasis [2,3]. Because LVI has not yet been incorporated into the TNM staging system, its prognostic relevance may be underestimated compared with more prominent factors such as lymph node metastasis and solid tumor size. However, Ma et al. reported that patients with small–sized lung adenocarcinomas with BVI who underwent wedge resection had poorer survival outcomes than those without BVI [13]. Furthermore, in this study, LVI was the strongest independent predictor of OS (OR: 3.850; 95%CI: 2.564–5.782; *p* < 0.001). These findings highlight the importance of preoperative prediction of LVI, which may contribute significantly to surgical decision–making for early–stage lung cancer.

Several studies have demonstrated the usefulness of the radiomics approach in identifying factors associated with poor prognosis and high recurrence rates in patients with early–stage lung cancer [16,28,29,30,31,32]. Cong et al. developed a radiomics model for predicting lymph node involvement in early–stage NSCLC, and the predictive performance of their radiomics model was significantly better than that of a model based on clinical factors alone [33]. A summary of artificial intelligence/radiomics studies predicting LVI or related pathological features in early–stage lung cancer is shown in Table 3. In our work, we reported that specific radiomics features were valuable for predicting early recurrence [16]. However, selectively focusing on a few parameters does not translate well into clinical practice because the process of obtaining these parameters is understood only by AI algorithms, leaving physicians unable to interpret the underlying mechanism. Therefore, we used a risk score derived from all 35 radiomics features after logit transformation. This was superior to using solid–part size, which is a well–known malignant indicator and the strongest clinical factor for LVI in this study. Although the presented AI–based radiomics model demonstrated high predictive performance, its generalizability to other institutions and imaging platforms remains to be verified. Differences in CT acquisition parameters, reconstruction algorithms, and image preprocessing may influence radiomic feature extraction and model performance. For clinical implementation, standardized imaging protocols and cross–platform harmonization will be essential. Moreover, external validation using independent, multicenter datasets is necessary to confirm the robustness, reproducibility, and clinical utility of the proposed model before widespread adoption.

EVs are nano–sized, membrane–bound vesicles secreted into the extracellular space [22,23,26]. Owing to their central role in cell–to–cell communication and their variable cargos, EVs are involved in cancer biological processes [22,23]. The ability to isolate EVs from various biofluids makes them valuable biomarkers for the diagnosis and prognosis of several conditions. In our previous study, miR–30d–5p levels were significantly downregulated in patients with LVI–positive lung adenocarcinoma, while miR–30d–5p levels in healthy donors were also lower than those in lung adenocarcinoma patients [21]. Additionally, patients with high miR–30d–5p levels had better survival rates compared to those with low miR–30d–5p levels [21]. Based on these findings, we concluded that miR–30d–5p levels in EVs may serve as a promising biomarker for detecting LVI in patients with early–stage lung adenocarcinoma.

Although there are still several hurdles to overcome before EVs can be used as clinical biomarkers, the integrated use of serum miR–30d levels and the risk score derived from AI radiomics analysis has demonstrated a very high accuracy for LVI, even in a limited number of patients with serum EV data. AI imaging and liquid biopsy are two promising, less invasive methods; when used together, they provide accurate and easy preoperative prediction of LVI. This advancement could become an important determinant of therapeutic decision–making within a multidisciplinary treatment process.

Our study had several limitations. First, it was a retrospective analysis conducted at a single institution, which may have introduced an inherent bias. Second, not all preoperative CT images could be successfully processed through the radiomics workflow; in some cases, a radiomics signature could not be generated due to low–fidelity CT images that could not be recognized by the AI system. Third, the AI algorithm used in this study is specific to the proprietary software platform used by our group, and applying these results to other centers would require the use of the same software. Consequently, the generalizability of the findings to other clinical settings or imaging systems may be limited. Variations in CT acquisition parameters, patient populations, and clinical practices across institutions could influence model performance. To enhance external validity, future studies should include multicenter datasets and prospective external validation. Fourth, the best method for isolating and characterizing EVs remains unclear. However, we have conducted several other studies using the same isolation methodology for serum–derived markers, supporting the validity of our approach. Fifth, another limitation of this study is that model calibration metrics, such as calibration plots and the Brier score, were not included in the current analysis. While the discrimination ability of the model was evaluated using the AUC, incorporating calibration metrics in future work will be important to provide a more comprehensive assessment of predictive performance and model reliability. Finally, another important limitation of this study is the relatively small number of cases with available serum EV–derived miR–30d data, which may limit the statistical power and lead to potential overestimation of the predictive performance. Variability in EV isolation methods, such as centrifugation conditions and extraction efficiency, may lead to differences in miRNA yield and quantification. In addition, the absence of external validation using datasets from other institutions restricts the generalizability of our findings. To strengthen the clinical applicability and reproducibility of the proposed model, future studies should include larger cohorts and prospective external validation across diverse imaging platforms and clinical environments.

## 5. Conclusions

In conclusion, this study demonstrated that AI–based radiomics analysis of preoperative CT scan is highly effective in predicting LVI in patients with early–stage lung adenocarcinoma. By using a large–scale imaging dataset and a standardized AI workflow, the model achieved robust performance and generated risk scores that may have broad clinical applications in preoperative risk stratification and individualized treatment planning. Furthermore, integrating radiomic features with serum EV–derived miR–30d levels further enhanced the predictive accuracy, suggesting the complementary value of imaging and liquid biopsy–based noninvasive approaches. By addressing a major gap in current diagnostic practice, these findings offer a novel direction for preoperative evaluation of LVI and advancing precision intervention strategies in early–stage lung cancer.

Future work will focus on validating this multimodal model in external and multicenter cohorts to assess its generalizability across diverse imaging platforms and patient populations. Additional studies are warranted to optimize biomarker integration, incorporate other molecular signatures, and refine the predictive model through advanced machine–learning techniques. Prospective trials will also be necessary to evaluate whether incorporating this risk score into surgical decision–making can improve long–term outcomes. Ultimately, expanding the model into a clinical deployable decision–support tool represents the next step toward establishing a noninvasive, precision–guided workflow for LVI assessment.

## Figures and Tables

**Table 1:** Patient characteristics.

Variable	Derivation Cohort n = 840 (%)	Validation Cohort n = 425 (%)	*p*
Age, years (median)	33–88 (68)	23–87 (68)	0.90
Sex, male	391 (47)	188 (44)	0.44
Any smoking history	442 (53)	225 (53)	0.95
Comorbidities, present	433 (52)	229 (54)	0.44
Whole tumor size on CT, cm (mean ± SD)	0.50–8.40 (2.20 ± 0.87)	0.50–5.00 (2.21 ± 0.88)	0.87
Solid tumor size on CT, cm (mean ± SD)	0.0–4.00 (1.54 ± 1.01)	0.0–4.00 (1.60 ± 1.06)	0.31
Clinical stage			0.12
0	75 (9)	28 (7)	
IA1	219 (26)	128 (30)	
IA2	271 (32)	123 (29)	
IA3	145 (17)	65 (15)	
IB	130 (15)	81 (19)	
Surgical procedure			0.32
Lobectomy	729 (87)	376 (88)	
Segmentectomy	75 (9)	38 (9)	
Wedge resection	36 (4)	11 (3)	
Pathological stage			0.041
0	13 (2)	8 (2)	
IA	571 (68)	295 (69)	
IB	162 (19)	58 (14)	
II	52 (6)	30 (7)	
III–IV	42 (5)	34 (8)	
Lymph–node status			0.038
N0	762 (91)	368 (87)	
N1	42 (5)	27 (6)	
N2	34 (4)	30 (7)	
Lymphovascular invasion, Positive	309 (37)	158 (37)	0.90
Blood vessel invasion, Positive	235 (37)	121 (28)	0.90
Lymphatic invasion, Positive	254 (30)	140 (33)	0.90
Collected serum extracellular vesicles	31 (4)	16 (4)	1.00

**Table 2:** The performance of the risk score in predicting lymphovascular invasion.

		Risk Score		AUC	Sens. (%)	Spec. (%)	Accu. (%)	PPV (%)	NPV (%)
	LVI	≤0.397	>0.397						
Derivation	Negative Positive	443	88	0.899	84.8	83.7	83.9	74.9	90.4
		47	262						
Validation	Negative Positive	212	55	0.882	82.3	79.4	80.5	70.3	88.3
		28	130						

**Table 3:** Summary of artificial intelligence/radiomics studies predicting lymphovascular invasion or related pathological features in early–stage lung cancer.

Study	Target Feature	Sample Size	Imaging Modality	AI/Radiomics Method	Key Finding
Chen et al. (2020) [28]	STAS	233	CT	Naïve Bayes model	AUC 0.63–0.69
Takehana et al. (2022) [34]	STAS	339	CT	Peritumoral features	AUC 0.70–0.76
Suh et al. (2024) [35]	STAS	520	CT	Radiomics score	AUC 0.815
Cong et al. (2020) [33]	LNM	649	CT	LASSO	AUC 0.851–0.898
Shimada et al. (2022) [16]	recurrence	642	CT	Modified U–Net	AUC 0.707–0.71
Wang et al. (2023) [36]	LVI	148	PET/CT	LASSO	AUC 0.773–0.774
Nie et al. (2021) [37]	LVI	272	PET/CT	Radiomics score	AUC 0.796–0.851
Chen et al. (2023) [38]	LVI	240	CT	Radiomics score	AUC 0.66–0.89
Lin et al. (2025) [39]	LVI	384	CT	Radiomics score	AUC 0.75–0.83
Present study	LVI	1265	CT	Modified U–Net	AUC 0.882–0.899

AI, artificial intelligence; STAS, spread through air spaces; CT, computed tomography; AUC, area under the curve; LNM, lymph node metastasis; LASSO, the least absolute shrinkage and selection operator; LVI, lymphovascular invasion; PET, positron emission tomography.

## Data Availability

The data used in this study are not publicly available due to patient privacy concerns and institutional restrictions but are available from the corresponding author upon reasonable request.

## Supplementary Materials

The following supporting information can be downloaded at: https://www.mdpi.com/article/10.3390/cancers17243998/s1, Figure S1. Logit transformation of the artificial intelligence score for lymphovascular invasion in 20 radiomics features for linearizing sigmoid distributions of proportions. Figure S2. (A) Overall survival curves of patients with lymphatic permeation positive and lymphatic permeation negative. (B) Recurrence–free survival curves of patients with lymphatic permeation positive and lymphatic permeation negative. (C) Overall survival curves of patients with blood vascular invasion positive and blood vascular invasion negative. (D) Recurrence–free survival curves of patients with blood vascular invasion positive and blood vascular invasion negative. Figure S3. Heat map of artificial intelligence radiomics features associated with the incidence of lymphovascular invasion in the derivation cohort. An annotations bar shows the presence or absence of lymphovascular invasion. Figure S4. Receiver–operating characteristics area under the curve (0.899, 95% confidence interval, 0.877 to 0.921, *p* < 0.001) for the risk score to identify lymphovascular invasion in the derivation cohort. Receiver–operating characteristics area under the curve (0.882, 95% confidence interval, 0.848 to 0.916, *p* < 0.001) for the rsik score to identify lymphovascular invasion in the validation cohort. Figure S5. Receiver–operating characteristics area under the curve (0.803, 95% confidence interval, 0.774 to 0.833, *p* < 0.001) for solid–part size to identify lymphovascular invasion in the derivation cohort. Figure S6. The relationship between extracellular vesicles–derived miR–30d level and the risk score in 47 patients who underwent liquid biopsy assessment, with or without lymphovascular invasion. Table S1. List of 20 radiomic features extracted using feature analysis model. Table S2. Univariate and multivariate analysis for overall survival. Table S3. Univariate and multivariate analysis for recurrence–free survival. Table S4. Univariate and multivariate logistic regression analysis to build the artificial intelligence score for predicting lymphovascular invasion. Table S5. Univariate and multivariate analysis for lymphovascular invasion in the derivation cohort. Table S6. The performance of solid–tumor size in predicting lymphovascular invasion. Table S7. The performance of extracellular vesicle–derived miR–30d level in predicting lymphovascular invasion in 47 patients who underwent liquid assessment. Table S8. The performance of the combined use of extracellular vesicle–derived miR–30d level and the risk score in predicting lymphovascular invasion in 47 patients who underwent liquid assessment.

## Abbreviations

The following abbreviations are used in this manuscript:

AI	artificial intelligence
AUC	area under the curve
CT	computed tomography
EVs	extracellular vesicles
LVI	lymphovascular invasion
miRNA	microRNA
NSCLC	non–small cell lung cancer
OS	overall survival
RFS	recurrent–free survival
ROC	receiver operating characteristic
