# Supplementary material for: Prediction of Lymphovascular Invasion in Early–Stage Lung Adenocarcinoma Using Artificial Intelligence–Based Radiomics
Source: Cancers (Basel). 2025 Dec 15;17(24):3998. doi: 10.3390/cancers17243998 (PMC12731777; doi:10.3390/cancers17243998)
Supplement: Supplementary file 1 [file cancers-17-03998-s001.zip › SUPPLEMENTARY FIGURE LEGENDS.pdf]

Supplementary Figure S1. Logit transformation of the artificial intelligence score for lymphovascular invasion in 20 radiomics features for linearizing sigmoid distributions of proportions.

Supplementary Figure S2. (**A**) Overall survival curves of patients with lymphatic permeation positive and lymphatic permeation negative. (**B**) Recurrence-free survival curves of patients with lymphatic permeation positive and lymphatic permeation negative. (**C**) Overall survival curves of patients with blood vascular invasion positive and blood vascular invasion negative. (**D**) Recurrence-free survival curves of patients with blood vascular invasion positive and blood vascular invasion negative.

Supplementary Figure S3. Heat map of artificial intelligence radiomics features associated with the incidence of lymphovascular invasion in the derivation cohort. An annotations bar shows the presence or absence of lymphovascular invasion.

Supplementary Figure S4. Receiver-operating characteristics area under the curve (0.899, 95% confidence interval, 0.877 to 0.921, $p < .001$) for the risk score to identify lymphovascular invasion in the derivation cohort. Receiver-operating characteristics area under the curve (0.882, 95% confidence interval, 0.848 to 0.916, $p < .001$) for the rsik score to identify lymphovascular invasion in the validation cohort.

Supplementary Figure S5. Receiver-operating characteristics area under the curve (0.803, 95% confidence interval, 0.774 to 0.833, $p < .001$) for solid-part size to identify lymphovascular invasion in the derivation cohort.

Supplementary Figure S6. The relationship between extracellular vesicles-derived miR-30d level and the risk score in 47 patients who underwent liquid biopsy assessment, with or without lymphovascular invasion.
