# Supplementary material for: Prediction of Lymphovascular Invasion in Early–Stage Lung Adenocarcinoma Using Artificial Intelligence–Based Radiomics
Source: Cancers (Basel). 2025 Dec 15;17(24):3998. doi: 10.3390/cancers17243998 (PMC12731777; doi:10.3390/cancers17243998)
Supplement: Supplementary file 1 [file cancers-17-03998-s001.zip › SUPPLEMENTARY TABLE.pdf]

Supplementary Table S1. List of 20 radiomic features extracted using feature analysis model.

Category	Class
Shape	Irregular
	Roundness
	smooth
Margin	Well defined
	Irregular edge
	Serrate
	Spicula
	Notch
	Polygons
Internal Characteristics	Bronchial translucency
	Cavity
	Calcification
	Fatness
External Characteristics	Pleural contaction
	Pleural indentation
	Broncho-vascular convergence
	Broncho-vascular compression
	Pleural recess
	Pleural hypertrophy
Opacity	Solid

Supplementary Table S2. Univariate and multivariate analysis for overall survival.

	Univariate analysis	
Variable	Odds ratio (95% confidence interval)	*P*
Age	1.027 (1.009 – 1.045)	0.003
Sex (male vs. female)	1.808 (1.310 – 2.494)	< 0.001
Smoking	1.823 (1.305 – 2.544)	< 0.001
Comorbidities	2.024 (1.451 – 2.824)	< 0.001
FEV1.0%	0.981 (0.964 – 0.997)	0.023
Solid-part size on computed tomography	1.485 (1.277 – 1.727)	< 0.001
Clinical stage (IB vs. 0-IA3)	1.413 (0.962 – 2.077)	0.078
Performance status (1-2 vs. 0)	1.323 (0.697 – 2.512)	0.392
Procedure (sublobar vs. lobectomy)	1.222 (0.771 – 1.938)	0.393
Pathological lymph node metastasis	5.150 (3.704 – 7.159)	< 0.001
Lymphovascular invasion	5.044 (3.519 – 7.229)	< 0.001
	Multivariate analysis	
	Odds ratio (95% confidence interval)	
Age	1.031 (1.011 – 1.051)	0.002
Smoking	1.774 (1.255 – 2.507)	0.001
Comorbidities	1.515 (1.061 – 2.164)	0.022
Procedure (sublobar vs. lobectomy)	1.880 (1.151 – 3.067)	0.012
Pathological lymph node metastasis	2.943 (2.034 – 4.258)	< 0.001
Lymphovascular invasion	3.850 (2.564 – 5.782)	< 0.001

FEV1.0%, forced expiratory volume in 1 second as a percentage of the forced vital capacity.

Supplementary Table S3. Univariate and multivariate analysis for recurrence-free survival.

	Univariate analysis	
Variable	Odds ratio (95% confidence interval)	*P*
Age	1.009 (0.996 – 1.022)	0.178
Sex (male vs. female)	1.714 (1.327 – 2.212)	< 0.001
Smoking	1.543 (1.190 – 2.001)	0.001
Comorbidities	1.617 (1.248 – 2.094)	< 0.001
FEV1.0%	0.989 (0.976 – 1.003)	0.126
Solid-part size on computed tomography	1.780 (1.581 – 2.004)	< 0.001
Clinical stage (IB vs. 0-IA3)	1.961 (1.469 – 2.617)	< 0.001
Performance status (1-2 vs. 0)	1.451 (0.886 – 2.376)	0.139
Procedure (sublobar vs. lobectomy)	1.370 (0.892 – 2.106)	0.151
Pathological lymph node metastasis	7.954 (6.107 – 10.361)	< 0.001
Lymphovascular invasion	6.629 (4.934 – 8.906)	< 0.001
	Multivariate analysis	
	Odds ratio (95% confidence interval)	
Sex	1.580 (1.221 – 2.045)	< 0.001
Solid-part size on computed tomography	1.501 (1.215 – 1.853)	< 0.001
Clinical stage (IB vs. 0-IA3)	1.445 (0.962 – 2.169)	0.076
Procedure (sublobar vs. lobectomy)	1.770 (1.105 – 2.833)	0.018
Pathological lymph node metastasis	3.579 (2.674 – 4.789)	< 0.001
Lymphovascular invasion	3.382 (2.369 – 4.827)	< 0.001

FEV1.0%, forced expiratory volume in 1 second as a percentage of the forced vital capacity.

Supplementary Table S4. Univariate and multivariate logistic regression analysis to build the artificial intelligence score for predicting lymphovascular invasion.

Variables	Univariate Odds ratio (95% CI)	*P*	Multivariate Odds ratio (95% CI)	*P*
V. Convergence	1.358 (1.240-1.487)	< 0.001	4.263 (2.313-7.858)	< 0.001
V. Compression	1.359 (1.180-1.566)	< 0.001	3.736 (1.937-7.204)	< 0.001
CT Value SD	1.008 (1.005-1.011)	< 0.001	1.010 (1.002–1.018)	0.018
Solid Area	1.009 (1.007–1.010)	< 0.001	1.002 (0.996–1.008)	0.595
Spicula	1.542 (1.433-1.659)	< 0.001	2.737 (1.036-7.232)	0.042
P. Contaction	1.072 (1.030-1.115)	< 0.001	1.122 (0.984-1.280)	0.086
Solid CT Value SD	1.008 (1.005-1.011)	< 0.001	0.991 (0.981–1.001)	0.072
P. Depression	1.346 (1.248-1.451)	< 0.001	0.657 (0.344-1.254)	0.203
Polygons	0.785 (0.702-0.877)	< 0.001	0.724 (0.491–1.069)	0.104
Notch	1.800 (1.636-1.982)	< 0.001	0.692 (0.427–1.121)	0.134
Roundness	0.798 (0.750-0.850)	< 0.001	1.632 (0.799-3.335)	0.179
Serrate	1.543 (1.444-1.649)	< 0.001	0.564 (0.263-1.210)	0.141
Solid Ratio	1.041 (1.036–1.047)	< 0.001	1.050 (1.022–1.079)	< 0.001
Irregular	1.269 (1.190-1.354)	< 0.001	1.382 (0.645–2.963)	0.405
Average Solid CT Value	1.011 (1.008-1.013)	< 0.001	1.006 (1.000-1.012)	0.050
Min CT Value	1.002 (1.001-1.003)	< 0.001	1.001 (0.998–1.004)	0.423

Min Solid CT Value	0.998 (0.998-0.999)	< 0.001	0.999 (0.997 – 1.001)	0.257
Well Defined	1.575 (1.458-1.700)	< 0.001	1.266 (0.696–2.303)	0.440
Tumor Volume	1.000 (1.000–1.000)	< 0.001	1.000 (1.000–1.000)	0.560
Smooth	0.844 (0.755-0.943)	< 0.001	1.090 (0.711-1.670)	0.694
Average CT Value	1.007 (1.006-1.008)	< 0.001	0.996 (0.990–1.001)	0.110
P. Hypertrophy	1.336 (1.243-1.435)	< 0.001	0.924 (0.618-1.382)	0.701
Solid Length	1.127 (1.105-1.148)	< 0.001	0.986 (0.916–1.061)	0.698
Cavity	1.151 (1.085-1.220)	< 0.001	0.958 (0.825–1.112)	0.575
Fatness	1.481 (1.296-1.692)	< 0.001	1.028 (0.856–1.234)	0.767
B. Translucency	0.910 (0.866-0.957)	< 0.001	1.027 (0.795–1.327)	0.839
Max Solid CT Value	1.003 (1.002–1.004)	< 0.001	0.996 (0.988–1.004)	0.318
Solid Volume	1.000 (1.000–1.001)	< 0.001	1.000 (1.000–1.000)	0.669
Solid Type	1.229 (1.194-1.264)	< 0.001	1.022 (0.873-1.195)	0.790
P. Indentation	1.225 (1.160-1.292)	< 0.001	1.062 (0.882-1.279)	0.526
Area	1.002 (1.002-1.003)	< 0.001	0.999 (0.995-1.003)	0.595
Max CT Value	1.003 (1.002–1.004)	< 0.001	1.003 (0.996–1.010)	0.445
Calcification	1.902 (1.639-2/206)	< 0.001	0.986 (0.821-1.184)	0.881
Length	1.064 (1.046-1.082)	< 0.001	0.992 (0.911–1.081)	0.856
Irregular edge	1.118 (1.058-1.181)	< 0.001	0.915 (0.485–1.726)	0.784

CI, confidence interval; V. Convergence, Vascular Convergence, V. Compression, Vascular Compression; CT, computed tomography; SD, standard deviation; P. Contaction, Pleural Contaction; P. Depression, Pleural Depression; P. Hypertrophy, Pleural Hypertrophy; B. Translucency, Bronchus Translucency; P. indentation; Pleural Indentation.

Supplementary Table S5. Univariate and multivariate analysis for lymphovascular invasion in the derivation cohort.

	Univariate analysis	
Variable	Odds ratio (95% confidence interval)	*P*
Age	0.992 (0.978 – 1.005)	0.234
Sex (male vs. female)	1.680 (1.266 – 2.229)	< 0.001
Smoking	1.694 (1.274 – 2.253)	< 0.001
Comorbidities	1.354 (1.021 – 1.794)	0.035
FEV1.0%	0.974 (0.958 – 0.990)	0.001
Solid-part size on computed tomography	3.415 (2.823 – 4.130)	< 0.001
Clinical stage (IB vs. 0-IA3)	3.936 (2.658 – 5.830)	< 0.001
Performance status (1-2 vs. 0)	1.524 (0.875 – 2.653)	0.136
Procedure (sublobar vs. lobectomy)	2.627 (1.610 – 4.287)	< 0.001
	Multivariate analysis	
	Odds ratio (95% confidence interval)	
Age	0.968 (0.951 – 0.986)	< 0.001
Sex (male vs. female)	1.479 (1.048 – 2.088)	0.026
FEV1.0%	0.976 (0.956 – 0.996)	0.017
Solid-part size on computed tomography	3.997 (3.159 – 5.058)	< 0.001
Clinical stage (IB vs. 0-IA3)	1.580 (0.915 – 2.725)	0.101

FEV1.0%, forced expiratory volume in 1 second as a percentage of the forced vital capacity.

Supplementary Table S6. The performance of solid-tumor size in predicting lymphovascular invasion.

	LVI	Solid tumor size		AUC	Sensitivity (%)	Specificity (%)	Accuracy (%)	PPV (%)	NPV (%)
		≤ 1.45cm	> 1.45cm						
Derivation	Negative	350	181	0.803	83.1	65.9	72.2	58.7	87.1
	Positive	52	257						
Validation	Negative	178	89	0.797	81.6	66.7	69.8	59.1	86.0
	Positive	29	129						

LVI, lymphovascular invasion; PPV, positive predictive value; NPV, negative predictive value.

Supplementary Table S7. The performance of extracellular vesicle-derived miR-30d level in predicting lymphovascular invasion in 47 patients who underwent liquid assessment.

LVI	miR-30d level		Sensitivity (%)	Specificity (%)	Accuracy (%)	PPV (%)	NPV (%)
	≤ 1.8	> 1.8					
Negative	3	14	70.0	82.4	74.5	87.5	60.9
Positive	21	9					

LVI, lymphovascular invasion; PPV, positive predictive value; NPV, negative predictive value.

Supplementary Table S8. The performance of the combined use of extracellular vesicle-derived miR-30d level and the risk score in predicting lymphovascular invasion in 47 patients who underwent liquid assessment.

LVI	miR-30d level + the risk score		Sensitivity (%)	Specificity (%)	Accuracy (%)	PPV (%)	NPV (%)
	> 1.8 or ≤ 0.397	≤ 1.8 or > 0.397					
Negative	12	5	93.3	70.5	85.1	84.8	85.7
Positive	2	28					
