# Supplementary figures and images for: Prediction of Lymphovascular Invasion in Early–Stage Lung Adenocarcinoma Using Artificial Intelligence–Based Radiomics
Source: Cancers (Basel). 2025 Dec 15;17(24):3998. doi: 10.3390/cancers17243998 (PMC12731777; doi:10.3390/cancers17243998)

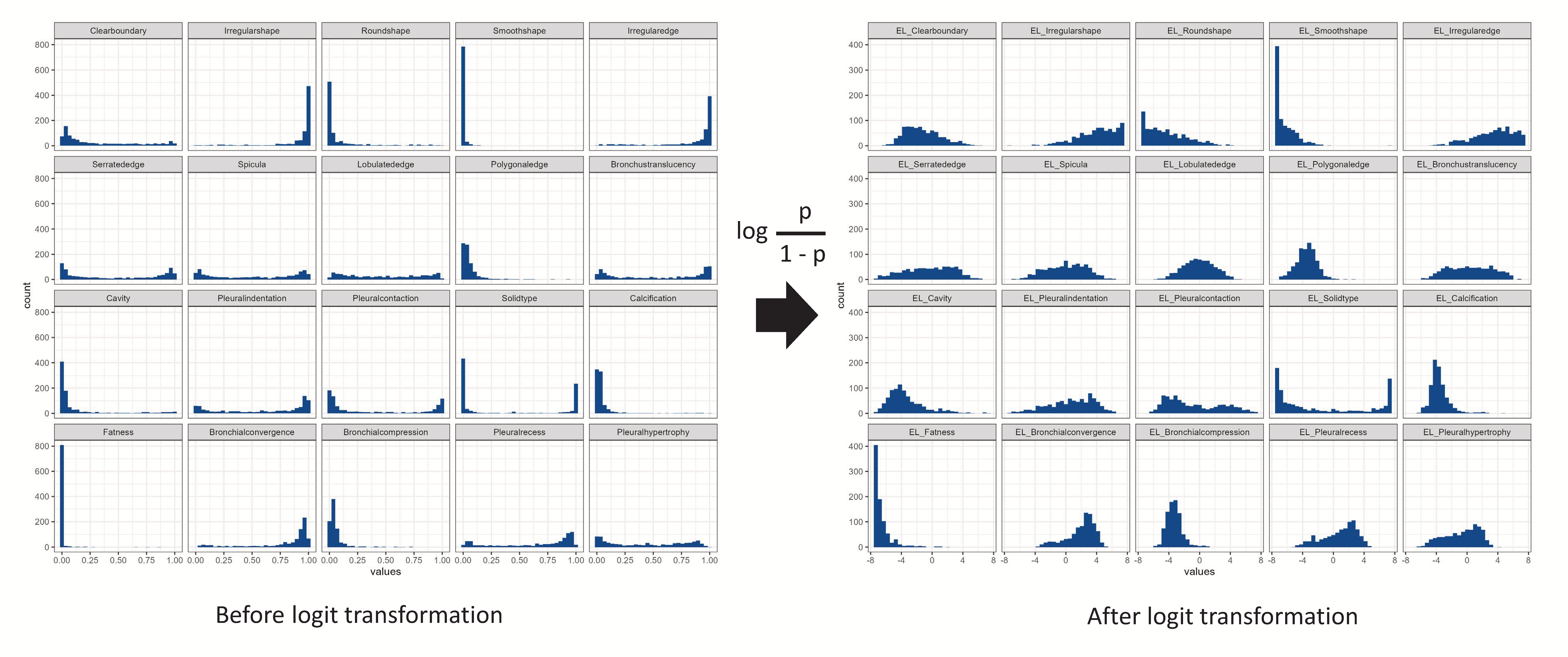

Supplement: Supplementary file 1 [file cancers-17-03998-s001.zip › Supplementary Figure S1.tif]

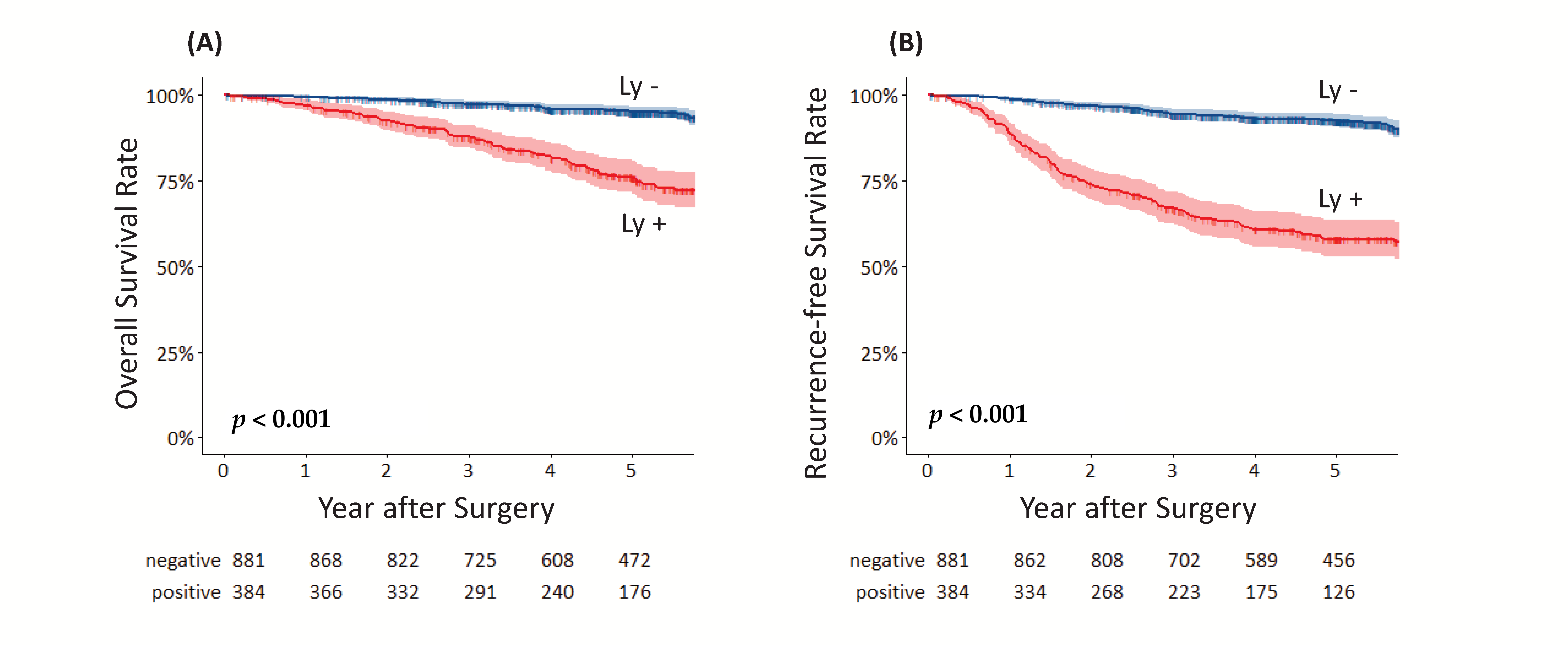

Supplement: Supplementary file 1 [file cancers-17-03998-s001.zip › Supplementary Figure S2-A,B.tif]

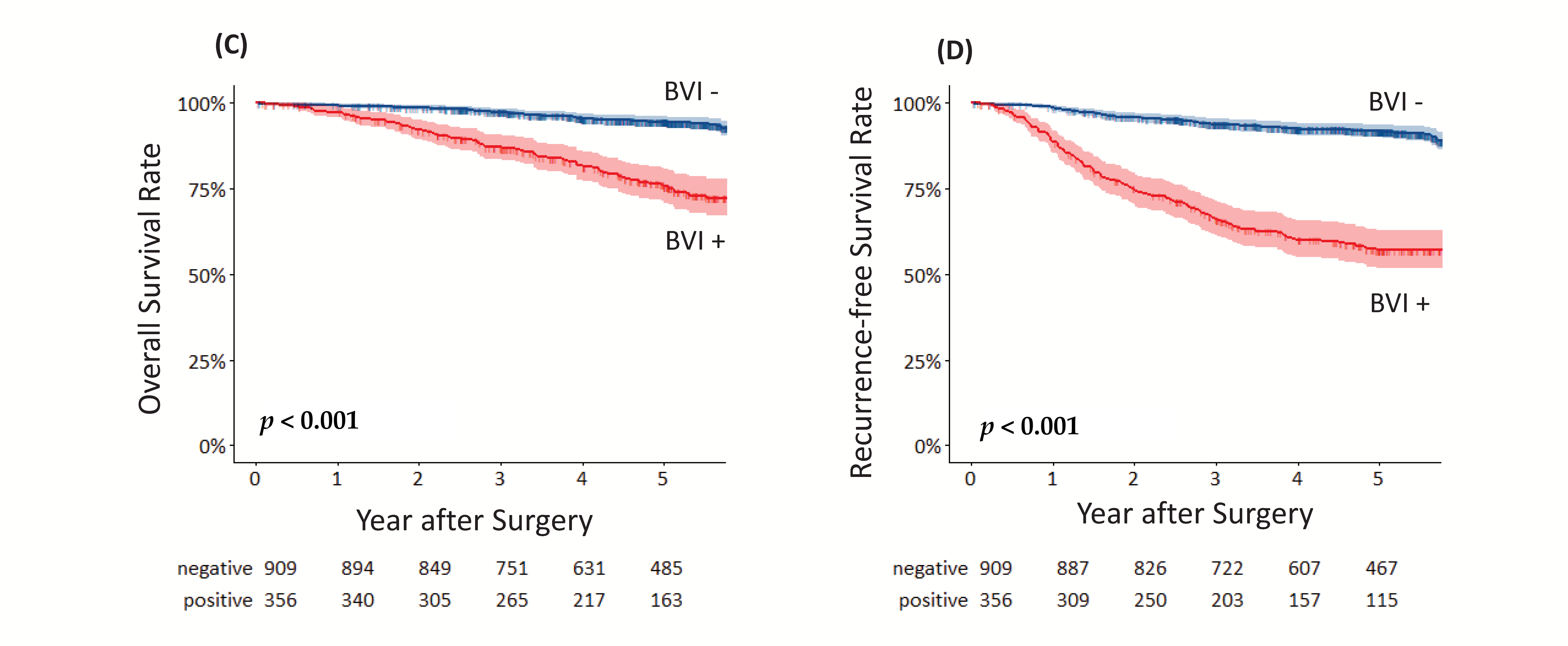

Supplement: Supplementary file 1 [file cancers-17-03998-s001.zip › Supplementary Figure S2-C,D.tif]

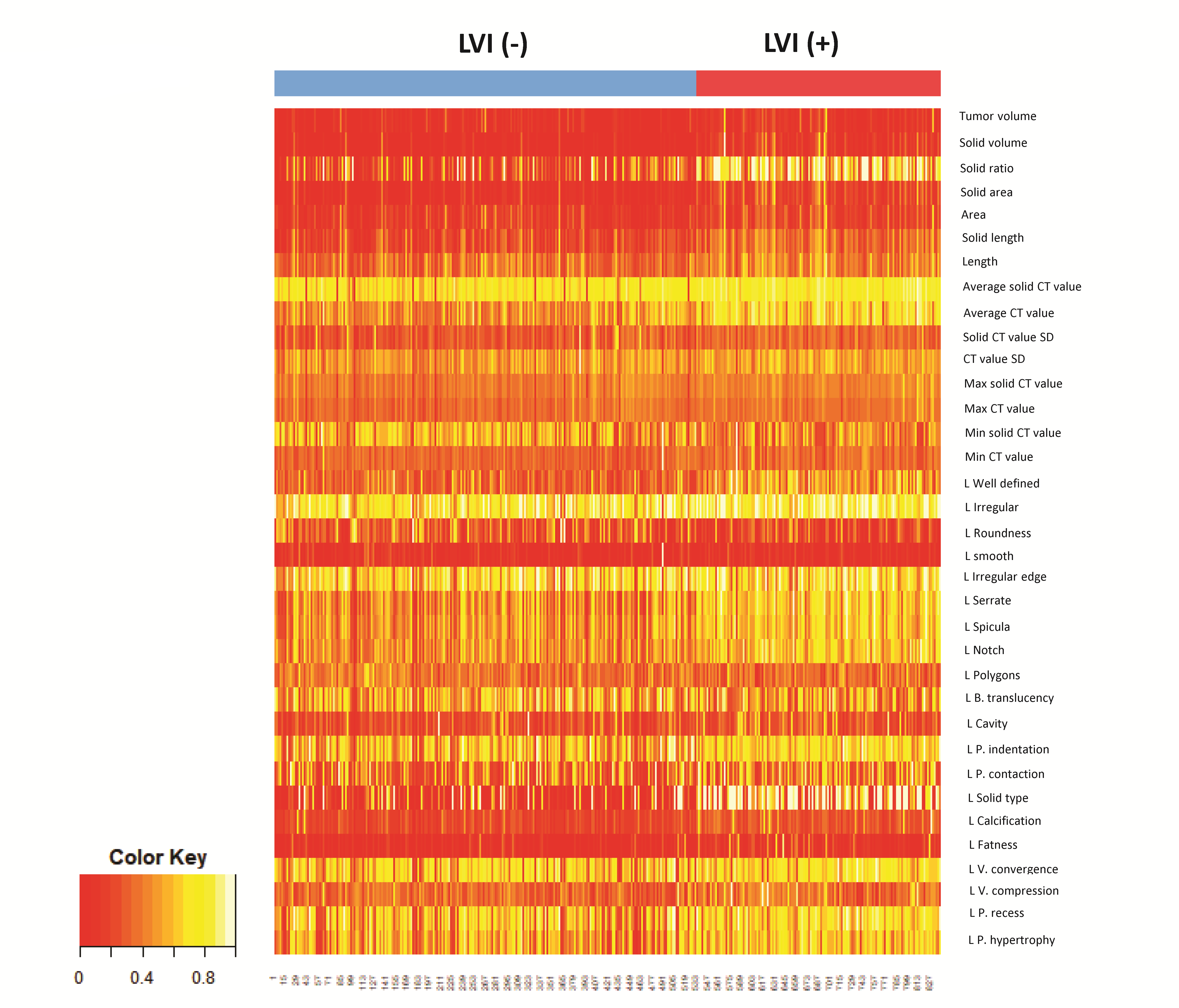

Supplement: Supplementary file 1 [file cancers-17-03998-s001.zip › Supplementary Figure S3.tif]

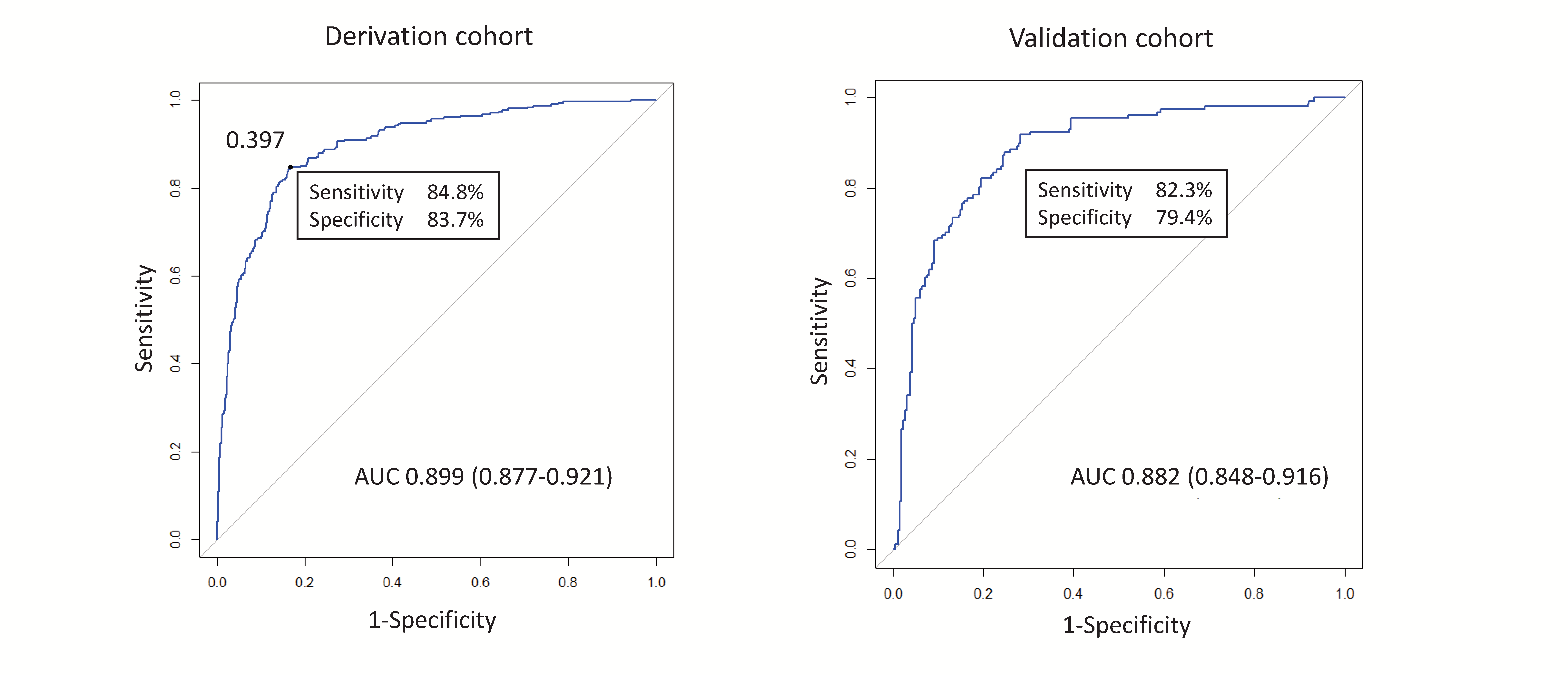

Supplement: Supplementary file 1 [file cancers-17-03998-s001.zip › Supplementary Figure S4.tif]

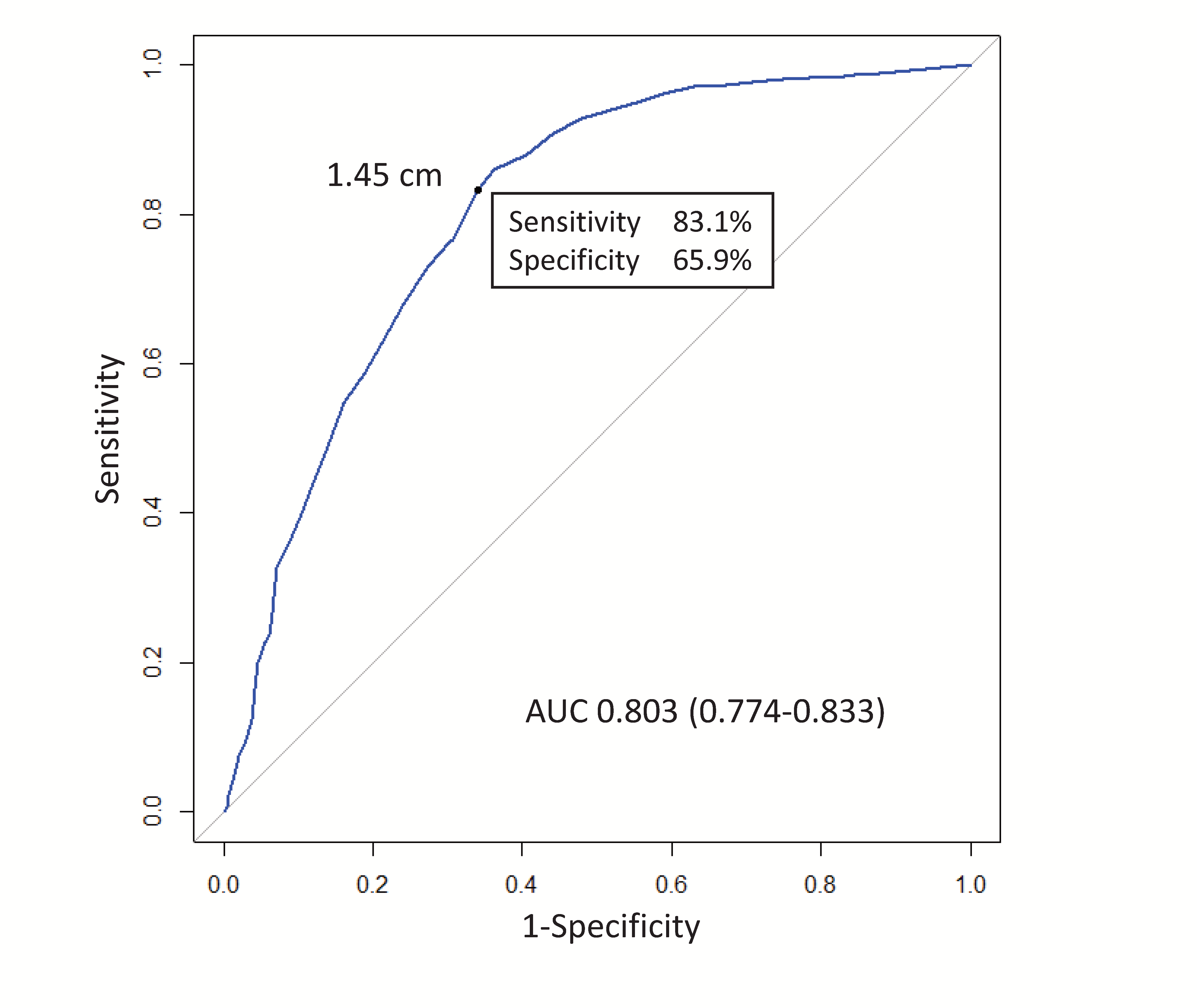

Supplement: Supplementary file 1 [file cancers-17-03998-s001.zip › Supplementary Figure S5.tif]

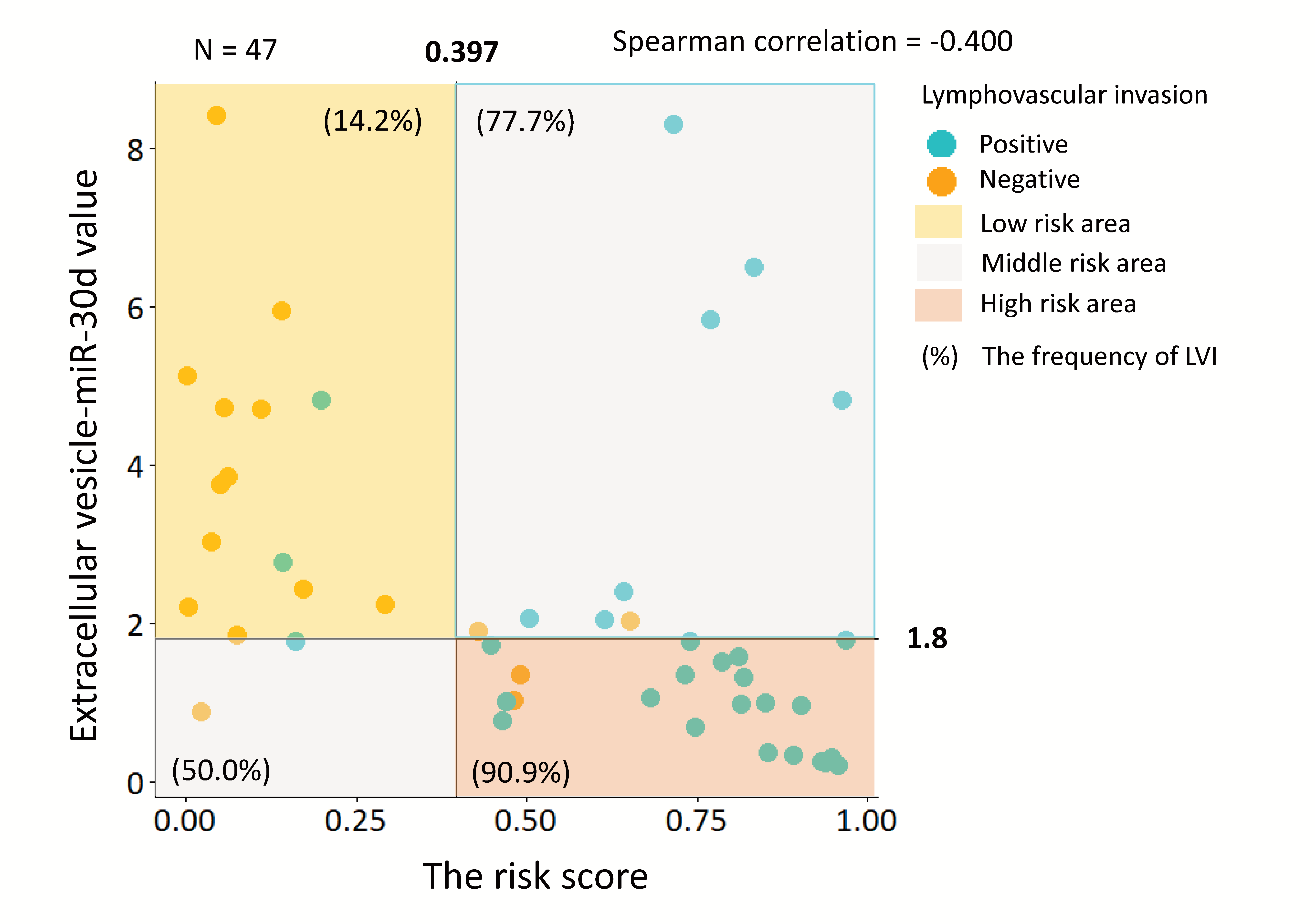

Supplement: Supplementary file 1 [file cancers-17-03998-s001.zip › Supplementary Figure S6.tif]
